# The role of histamine in narcolepsy type 1: a narrative review

**DOI:** 10.3389/fneur.2026.1900892

**Published:** 2026-10-06

**Authors:** Wei Liang, Xue Yan, Yanan Zhao, Dongnan Zhang, Yu Fu, Guifeng Sui, Weimin Zhang

**Affiliations:** 1 School of Traditional Chinese Medicine, Changchun University of Chinese Medicine, Changchun, China; 2 Brain Disease Center, The Third Affiliated Clinical Hospital of Changchun University of Chinese Medicine, Changchun, China; 3 Rehabilitation Department, Brain Disease Center, The Third Affiliated Clinical Hospital of Changchun University of Chinese Medicine, Changchun, China

**Keywords:** excessive daytime sleepiness, H1 receptor, H3 receptor, histamine, narcolepsy type 1, orexin, tuberomammillary nucleus

## Abstract

Narcolepsy type 1 (NT1) is a chronic neurological disorder characterized mainly by impaired wakefulness maintenance and dysregulated rapid eye movement (REM) sleep, which markedly affects patients’ daytime functioning and quality of life. The core pathological basis of NT1 is the selective loss of orexinergic neurons. The orexinergic system regulates histaminergic neurons in the tuberomammillary nucleus (TMN) through anatomical projections and functional excitation, while the histaminergic system contributes to cortical activation, arousal maintenance, and attention regulation. Therefore, the orexinergic and histaminergic systems can be regarded as interconnected and functionally complementary components of the arousal network. Previous studies have suggested alterations in histaminergic signaling in NT1, although the specific mechanisms underlying its role in NT1 remain incompletely understood. Current evidence suggests that histamine may play a modulatory role in NT1-associated phenotypes. This review summarizes the physiological functions of the histaminergic system in arousal regulation and discusses its interaction with the orexinergic system. It further analyzes the role of HA in NT1, its influence on clinical phenotypes, and related therapeutic strategies, with the aim of providing insights for mechanistic research and precision treatment of NT1.

## 1 Introduction

Narcolepsy is a rare and debilitating chronic neurological disorder. Epidemiological studies indicate that approximately 42 out of every 100,000 people are affected (1). The onset of narcolepsy follows a bimodal age distribution, with the first peak at around 15 years of age (adolescence) and the second peak between 30 and 39 years of age (adulthood) (2, 3). Based on clinical manifestations, orexin (ORX) levels in cerebrospinal fluid (CSF), and the presence or absence of cataplexy, narcolepsy can be classified into narcolepsy type 1 (NT1) and narcolepsy type 2 (NT2) (4, 5). Clinical manifestations vary greatly among patients. Some patients experience only one or a few of the following symptoms: excessive daytime sleepiness (EDS), disrupted nighttime sleep (DNS), sleep paralysis, hypnagogic/hypnopompic hallucinations, and cataplexy, and may also experience accompanying symptoms, including cognitive impairment, psychiatric disorders, and autonomic symptoms (6, 7). Pediatric patients often exhibit relatively pronounced, age-specific clinical manifestations in the early stages of the disease, including atypical cataplexy and motor control impairments, behavioral and neuropsychiatric symptoms, obesity, rapid weight gain, and metabolic disturbances (8, 9).

The selective loss of hypothalamic orexinergic neurons is a core pathological feature of NT1 (10, 11). Autopsy studies of patients with NT1 have revealed a marked reduction in hypothalamic orexinergic neurons and a widespread deficiency of ORX peptides in the brain, providing direct neuropathological evidence of profound disruption to the orexin system in human NT1 (11, 12). Animal model studies have shown that hereditary canine narcolepsy caused by mutations in the orexin receptor 2 (OX2R) gene, prepro-orexin knockout (KO) mice, and mice with ablated orexinergic neurons all exhibit unstable sleep–wake states and a narcolepsy-like phenotype (13–15). It is important to note that animal models and sporadic human NT1 arise from different biological contexts and should be viewed as convergent evidence supporting the importance of the orexinergic system; they should not be considered entirely equivalent pathological processes.

However, the mechanisms underlying this selective neuronal loss have not been fully established. Currently, a substantial body of evidence suggests that the selective loss of orexinergic neurons in NT1 may be associated with an immune-mediated process. This evidence includes the strong association between NT1 and HLA-DQB1*06:02, the increased incidence following the 2009 H1N1 influenza pandemic, and reports of autoreactive T cells targeting antigens associated with orexinergic neurons (16–18). However, direct evidence in the human hypothalamus showing that immune responses lead to the loss of orexinergic neurons remains limited.

Irrespective of the precise mechanism responsible for orexinergic neuronal loss, its consequences are likely to extend beyond the orexin system itself. Orexinergic neurons project widely to multiple brain regions involved in the regulation of arousal, motor function, emotion, autonomic nervous system activity, and rapid eye movement (REM) sleep, including the locus coeruleus, dorsal raphe nucleus, tuberomammillary nucleus (TMN), basal forebrain, thalamus, brainstem, and spinal cord (19–21). Therefore, the orexinergic system can be understood as a stabilizer of the sleep–wake cycle and an upstream integrator of the arousal network, rather than simply as a neurotransmitter system that increases wakefulness.

Histamine (HA) is also a key neurotransmitter involved in promoting and maintaining wakefulness, and it participates in the regulation of the sleep–wake cycle (22–24). However, previous findings regarding HA changes in NT1 have been inconsistent, and the biological implications suggested by different lines of evidence also vary (25–31).

Previous reviews have explored the regulatory effects of HA on wakefulness, receptor signaling, and the efficacy of related medications. However, most have discussed these topics within broader frameworks of sleep–wake regulation, narcolepsy diagnosis and treatment, or histaminergic pharmacology. Few reviews have systematically examined how histaminergic function is altered in the context of orexinergic neuron loss and how such changes may influence NT1 phenotypes. This narrative review integrates findings from human CSF studies, postmortem and histological evidence, animal models, and pharmacological studies, with particular attention to areas in which different lines of evidence converge or remain inconsistent. Building on this framework, this review provides a comprehensive assessment of the role of the histaminergic system in NT1 and its therapeutic relevance, offering guidance for future mechanistic research and the optimization of treatment strategies.

## 2 Literature search methods

This study used a mechanistic narrative review approach involving literature search and data synthesis to summarize basic research, clinical observations, and therapeutic evidence regarding HA in NT1. The literature search was conducted in PubMed and Web of Science Core Collection. The search period ranged from database inception to February 2026. Search terms included: “narcolepsy type 1,” “narcolepsy with cataplexy,” “orexin,” “histamine,” “cerebrospinal fluid histamine,” “H1 receptor,” “H3 receptor,” “pitolisant,” “OX2R agonist,” and related terms.

## 3 The role of HA in the regulation of arousal

### 3.1 Anatomical and electrophysiological basis of the histaminergic system

HA is a monoamine neurotransmitter synthesized when L-histidine is taken up by neurons via L-amino acid transporters and then decarboxylated by the specific enzyme L-histidine decarboxylase (HDC) (32–35). Unlike peripheral HA, which is mainly involved in immunity, inflammation, and gastric acid secretion, central HA is primarily involved in arousal, attention, cognition, energy metabolism, and autonomic regulation (33, 36, 37). In mammals, histaminergic neurons in the TMN of the posterior hypothalamus are the sole neuronal source of HA in the central nervous system (38, 39). TMN histaminergic neurons project diffusely to regions such as the cerebral cortex, thalamus, basal forebrain, hippocampus, amygdala, hypothalamus, brainstem, and spinal cord, forming a highly divergent arousal-regulating network (22, 40).

Histaminergic neurons show firing patterns that change dynamically across the sleep–wake cycle. During wakefulness, they maintain a low-frequency tonic discharge; this activity increases when the individual is actively alert, exploring the environment, or highly focused. During non-rapid eye movement (NREM) sleep, the firing frequency decreases, and during REM sleep, firing essentially ceases (33, 41). This discharge pattern, which is closely synchronized with sleep–wake states, provides electrophysiological evidence that the histaminergic system is a key pathway regulating arousal level.

### 3.2 H1 and H3 receptors in arousal regulation

In rodents, the synthesis, release, and metabolism of HA show circadian fluctuations, suggesting that HA can activate HA receptors in the brain to regulate behavioral activity and cortical arousal (34, 42). The arousal-related effects of HA are mainly mediated by two subtypes of G protein-coupled receptors: the H1 receptor (H1R) and the H3 receptor (H3R).

H1R is widely distributed in key brain regions involved in wakefulness regulation, with high density in the thalamus, cerebral cortex, and limbic system, and lower density in the spinal cord and cerebellum (40). Early studies found that administering the first-generation antihistamine promethazine to cats suppressed REM sleep and increased slow-wave sleep (SWS) (43). Similar findings have also been reported in laboratory dogs and human subjects (44, 45).

Further studies showed that direct intracerebroventricular injection of HA into anesthetized rats shortened the duration of anesthesia (46). These findings suggest that the sedative effects of H1-antihistamines may result from blockade of the wake-promoting effects of the histaminergic system. Consistent with this wake-promoting effect, knockout studies have further confirmed the central role of H1R. Knockout studies have further confirmed the central role of H1R. H1R-KO mice showed a significant reduction in wakefulness duration during the nocturnal active period. Cortical electroencephalographic (EEG) recordings showed a decreased power ratio between SWS and wakefulness. When exposed to external behavioral stimuli, these mice had shorter sleep latency and difficulty maintaining stable wakefulness (47–49). Human studies also support these findings: H1R antagonists reduce alertness and impair executive function, which is consistent with the sedative and somnolent effects caused by classic antihistamines and provides clinical evidence for the physiological significance of H1R-mediated arousal (50–52).

H3R functions as both an autoreceptor and a heteroreceptor, and is widely localized in the cell bodies, dendrites, and axon terminals of HA and other neurons (53, 54). The H3R is mainly located in presynaptic regions of histaminergic neurons and can regulate HA synthesis and release through negative feedback pathways (55, 56). H3 heteroreceptors suppress the release of several wake-promoting neurotransmitters, including acetylcholine, norepinephrine, and glutamate (57). Animal studies have shown that oral administration of H3R agonists significantly increases the proportion of SWS, whereas systemic administration or regional brain injection of H3R antagonists prolongs wakefulness and reduces SWS and REM sleep in a dose-dependent manner (58, 59).

Inhibition of the presynaptic H3 autoreceptors and heteroreceptors upregulates the release of several neurotransmitters, including HA and norepinephrine, that are involved in maintaining wakefulness and regulating cognition (60–62). This effect has shown potential therapeutic value in conditions such as NT1, cataplexy, Parkinson’s disease, attention-deficit/hyperactivity disorder, Alzheimer’s disease, and dementia (63, 64).

## 4 Anatomical and functional interactions between ORX and HA

Anatomical studies suggest reciprocal connections between the orexinergic and histaminergic systems. Histaminergic axons innervate orexinergic neurons in the lateral hypothalamic area. Immunoelectron microscopy further confirmed clear synaptic connections between orexinergic nerve terminals and HDC-positive cell bodies and dendrites. Orexinergic neurons form direct synaptic connections with histaminergic neurons in the TMN by releasing ORX peptides, thereby acting on OX2R (65). These experiments demonstrated, at the morphological level, the existence of a direct monosynaptic connection between the orexinergic and histaminergic systems. Eriksson et al. (66) found that orexinergic neurons receive extensive innervation from histaminergic axons, providing preliminary light-microscopic evidence for reciprocal projections. However, compared with the well-established orexinergic projections to histaminergic neurons, evidence for histaminergic projections to orexinergic neurons remains less direct and is mainly based on light-microscopic observations and cross-species morphological validation. Direct evidence from independent tracing and ultrastructural synaptic analyses remains to be further established.

At the functional level, electrophysiological experiments have shown that ORX induces membrane depolarization in histaminergic neurons in the TMN, mainly through OX2R-mediated activation of Na+/Ca2 + exchanger currents and Ca2 + influx. These findings provide direct functional evidence that ORX peptides potently excite histaminergic neurons (66). Optogenetic studies combined with brain-slice patch-clamp techniques further demonstrated that orexinergic neurons provide rapid glutamatergic transmission to histaminergic neurons, suggesting that the orexinergic system can dynamically regulate histaminergic arousal circuits (67). Behavioral experiments showed that intracerebral injection of orexin-A (ORX-A) into the TMN of rats leads to a dose-dependent increase in HA release in the medial preoptic area and prefrontal cortex, as well as an increase in wakefulness. However, in H1R-KO conditions, the sleep–wake rhythm remained unchanged, suggesting that the arousal-promoting effect of ORX-A is mediated, at least in part, by activation of histaminergic neurons and subsequent H1R signaling (68, 69).Mice in which OX2R expression was restored only in the TMN or basal forebrain showed significantly prolonged wakefulness and an increased number of long wakefulness episodes; however, cataplexy did not improve. In contrast, mice in which OX2R expression was restored only in the ventrolateral periaqueductal gray/lateral pontine tegmentum showed complete suppression of cataplexy but no significant change in wakefulness (70).

HA may also act through H1R to indirectly regulate the orexinergic system via a positive feedback mechanism. Experiments using zebrafish models have shown that blocking H1R reduces the development and formation of orexinergic neurons (71). In adult mammals, activation of H1R enhances the activity of upstream systems such as basal forebrain cholinergic and glutamatergic systems, thereby indirectly stimulating orexinergic neurons (72).

Based on the available anatomical and functional evidence, the orexinergic system regulates the activity of histaminergic neurons through direct synaptic connections. Although the histaminergic system can, in turn, influence the arousal network through feedback mechanisms, current evidence remains insufficient to establish an equivalent reciprocal regulation between the two systems. Therefore, these findings do not yet support characterizing the orexinergic and histaminergic systems as a closed-loop circuit. It is more appropriate to view the two as interconnected, functionally complementary subsystems within the arousal network, rather than as a simple upstream-downstream relationship (73) (Figure 1).

**Figure 1:**
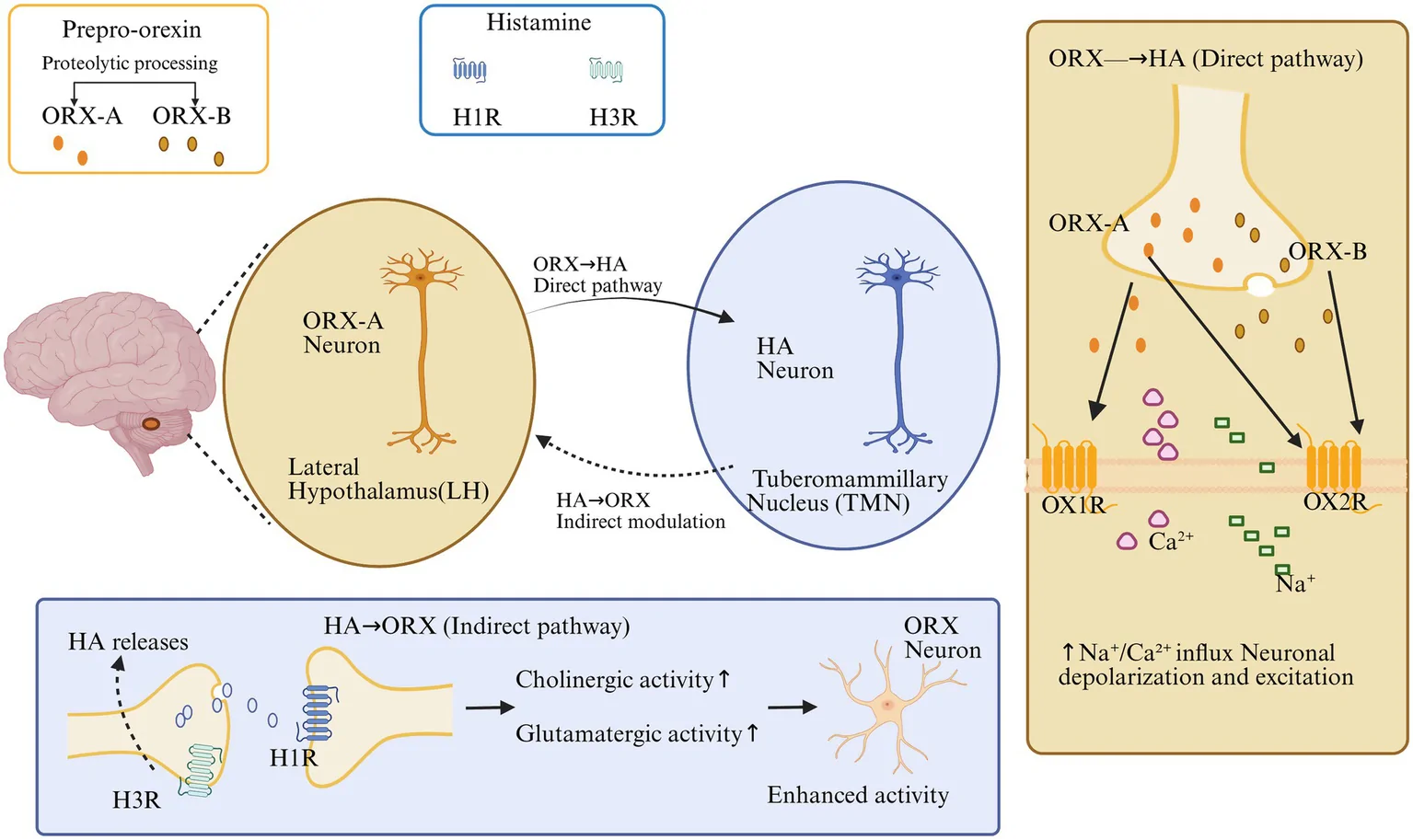
Schematic representation of the interaction between ORX and HA neurons. Prepro-orexin in the lateral hypothalamus is cleaved by orexin neurons to generate ORX-A and ORX-B. ORX-A activates OX1R and OX2R with roughly equal potency, whereas ORX-B preferentially activates OX2R, leading to increased intracellular calcium concentration and subsequent excitation of TMN histaminergic neurons. HA exerts its effects via G-protein-coupled receptors (GPCR) subtypes, H1R and H3R. H1R is a postsynaptic receptor whose activation enhances the activity of upstream pathways including basal forebrain cholinergic and glutamatergic systems, thereby indirectly modulating ORX neurons.

## 5 The relationship between HA and NT1

### 5.1 Human CSF evidence: inconsistent HA/ tele-methylhistamine(t-MH) findings

Early studies suggested that patients with narcolepsy and other central disorders of hypersomnolence may have reduced HA levels in CSF. Nishino et al. (25) first demonstrated graded differences in CSF HA levels: CSF HA concentrations were lowest in the low-CSF-ORX group, intermediate in narcolepsy patients with normal CSF ORX levels, and highest in healthy controls. Furthermore, the reduction in CSF HA levels was more pronounced in patients who were not receiving medication. Kanbayashi et al. (26) reported similar findings in a larger clinical study, showing that CSF HA levels were significantly reduced in patients with central hypersomnia, including narcolepsy and idiopathic hypersomnia (IH). Bassetti et al. (27) found that CSF HA concentrations were significantly lower in patients with EDS than in controls without sleepiness symptoms. Correlation analysis further showed a significant negative correlation between CSF HA levels and Epworth Sleepiness Scale scores.

However, subsequent studies did not consistently support the finding that CSF HA levels are reduced. Croyal et al. (28) measured the levels of HA and its metabolite t-MH in the CSF of seven patients with narcolepsy with cataplexy (NC) and found no statistically significant difference compared with controls. Dauvilliers et al. (74) found no statistically significant differences in CSF HA or t-MH levels across central hypersomnia subgroups. Neither measure showed a consistent association with sleepiness severity, medication history, or cataplexy frequency. These studies challenged the previous view that CSF HA levels are reduced in patients with central hypersomnia. Lopez et al. (29) further showed through longitudinal follow-up that markers of HA metabolism do not necessarily change in parallel with impairment of the orexinergic system.

Given the varying results reported in previous adult studies, research in pediatric patients with NT1 provides an additional perspective. Franco et al. (75) found that in children with ORX-deficient NT1, CSF HA concentrations were significantly elevated, t-MH concentrations were reduced, and the t-MH/HA ratio was markedly decreased. This biochemical profile is thought to reflect decreased HA turnover and impaired histaminergic neurotransmission and might support the hypothesis that age-related heterogeneity contributes to the variability and discrepancies observed across adult studies.

### 5.2 Postmortem and histological evidence: histaminergic neuronal changes

Unlike studies based on cerebrospinal fluid testing, autopsy and histological analyses mainly examine changes in the histaminergic system at the levels of cell number and tissue structure.

John et al. (76) reported increased histaminergic neurons in postmortem specimens from patients with NC, whereas HDC-positive neurons were unchanged in animal models. Valko et al. (77) reported an increased number of histaminergic neurons in postmortem samples from patients with narcolepsy and in ORX-KO mice. This increase appeared to be associated with the extent of orexinergic neuronal loss, and the increased number of histaminergic neurons may influence the symptoms and clinical management of narcolepsy. Berteotti et al. (78) showed in animal models that ORX peptide deficiency may increase the number of histaminergic neurons. However, HA deficiency does not appear to significantly affect the number of orexinergic neurons in mammals.

### 5.3 Animal model evidence: HA as a phenotype-modulating factor

Compared with postmortem and tissue-level studies, animal models offer an experimental approach to examining the functional consequences of histaminergic alterations in ORX deficiency.

In contrast to studies suggesting histaminergic neuronal expansion after ORX deficiency, Melzi et al. (30) found no clear compensatory upregulation of the histaminergic system in mouse models of congenital ORX deficiency or immune-mediated orexinergic neuronal injury. Bastianini et al. (31) suggested that HA deficiency primarily affects metabolic regulation and sleep-related breathing, whereas an intact histaminergic system is not required for the development of core narcolepsy symptoms, and HA deficiency alone cannot directly induce these symptoms.

### 5.4 Critical interpretation of conflicting evidence

As summarized in Table 1, previous studies have reached inconsistent conclusions on whether HA-related markers decrease, remain unchanged, or increase. The above experimental and clinical evidence indicates that there is no simple linear relationship between the histaminergic system and NT1. These discrepancies cannot be understood solely based on the direction of the results. Instead, they need to be considered in relation to the characteristics of the study population, disease stage, medication exposure, sampling conditions, measured markers, and the biological level of evidence.

**Table 1:** Study-level evidence framework for histaminergic alterations in NT1.

Evidence category	Study	Study subjects/models	Related measure	Main findings	Main limitation	Interpretation	References
Human CSF evidence	Nishino et al.	Low-CSF-ORX narcolepsy (*n* = 34), normal-CSF-ORX narcolepsy ( = 24), and healthy controls (n = 23).	CSF HA	Graded CSF HA decrease: low-ORX narcolepsy < normal-ORX narcolepsy < controls.	CSF sampling varied from 09:00 to 17:00; medication and pre-sampling sleep/activity were incompletely controlled.	Reduced CSF HA may be a marker or partial mediator of EDS.	(25)
	Kanbayashi et al.	Narcolepsy (*n* = 67), IH (*n* = 26), OSA (*n* = 16), and healthy controls (*n* = 73).	CSF HA	CSF HA was reduced in central hypersomnia conditions, including narcolepsy and IH.	No explicit limitation identified in the available text; cross-sectional CSF measurement may limit dynamic interpretation.	CSF HA may reflect central hypersomnia severity, but lacks NT1 specificity.	(26)
	Bassetti et al.	EDS status, including patients with narcolepsy, IH, and OSA (*n* = 28).	CSF HA	Lower CSF HA in sleepy patients; negative correlation with ESS scores.	Small sample; inconsistent lumbar puncture timing; medication use and age/sex matching not fully controlled.	Low CSF HA may reflect broader arousal-network and inflammatory-system interactions.	(27)
	Croyal et al.	NC (*n* = 7) and controls (*n* = 32).	CSF HA and t-MH	No significant group differences in CSF HA or t-MH.	Methodological issues and small sample size may underlie discrepancies with prior studies.	Challenges the view that reduced CSF HA is a consistent feature of narcolepsy with cataplexy.	(28)
	Dauvilliers et al.	NC (*n* = 56), NwC (*n* = 27), IH (*n* = 11), secondary narcolepsy (*n* = 3), unspecified hypersomnia Uns EDS (*n* = 17) and Controls (*n* = 50).	CSF ORX-A, HA, and t-MH	No association was found between CSF HA, t-MH, sleepiness, treatment intake, and frequency of cataplexy.	Neurological controls; EDS/pre-puncture sleep fluctuations; lumbar CSF HA/t-MH may poorly index brain HA release.	CSF HA/t-MH are not informative for differentiating central hypersomnia etiologies or assessing severity.	(74)
	Lopez et al.	HLA-DQB1*06:02-positive primary narcolepsy with definite/atypical cataplexy (*n* = 170); repeat CSF ORX and HA/t-MH in selected patients.	Longitudinal CSF ORX, HA, and t-MH	CSF ORX changes were not linked to demographic/clinical features.	CSF assay variability, heterogeneous sampling intervals, and imprecise cataplexy classification based on clinical history.	Cataplexy may occur before CSF ORX deficiency, indicating heterogeneous ORX neuron loss and supporting repeat CSF biomarker assessment.	(29)
	Franco et al.	NT1 children (*n* = 24) and controls (*n* = 21).	CSF HA and t-MH	Increased CSF HA and decreased t-MH levels, with reduced t-MH/HA ratios in NT1 children.	Small sample; patient heterogeneity; limited control selection; insufficient assessment of sleep status; potential confounding factors in CSF measurements	Decreased HA turnover and impaired histaminergic neurotransmission, supporting histaminergic therapy.	(75)
Human postmortem / tissue-level evidence	John et al.	Narcoleptic human brains (*n* = 5) and controls (*n* = 7). OX2R-mutant narcoleptic Dobermans (*n* = 3) and matched controls (*n* = 3). HDC-KO mice (*n* = 4) and WT controls (*n* = 4). ORX-KO and WT mice (age-matched littermate males; *n* = 12). Ataxin-3-ORX mice and age-matched littermate controls (*n* = 24). DTA-ORX mice with postnatal ORX-neuron ablation and controls (*n* = 10).	HDC-immunoreactive histaminergic neurons	HDC-labeled cells increased in human narcolepsy, but not in animal models versus their controls.	Potential medication effects; all human cases had stimulant/antidepressant exposure, without corresponding animal validation.	Human narcolepsy may involve expansion of histaminergic containing cells.	(76)
	Valko et al.	Human narcolepsy patients (*n* = 7) and controls (*n* = 12); WT mice (*n* = 6), orexin/ataxin-3 mice (*n* = 6), and ORX ligand-KO mice (*n* = 5).	Stereological counts of ORX, MCH, and histaminergic neurons	Histaminergic neurons increased in human narcolepsy, especially with severe ORX-neuron loss; ORX-KO mice also showed increased histaminergic neurons.	Potential HDC antibody cross-reactivity and postmortem-delay differences may affect HDC immunoreactivity.	May reflect compensatory HA remodeling after ORX loss, with potential symptom and therapeutic relevance.	(77)
	Berteotti et al.	Adult male ORX-KO, HDC-KO, and WT mice (*n* = 4/group).	Counts of hypothalamic HDC-positive histaminergic neurons and ORX-A-positive neurons.	Orexin-knockout mice had more HDC-positive neurons than wild-type mice, whereas HDC-knockout mice did not show a significant reduction in orexinergic neurons.	Small sample size, reliance on immunohistochemistry, possible HDC-antibody cross-reactivity, and no direct brain HA measurement.	ORX loss may drive histaminergic neuron expansion, but HA loss does not impair orexinergic neuron development.	(78)
Animal tissue-level evidence	Melzi et al.	WT mice (*n* = 5), ORX-KO mice (*n* = 5), ORX-HA no-T (with no T cells transfer) mice (*n* = 4), ORX-HA-2xCD8 mice (*n* = 6), and HDC-KO mice for antibody validation (*n* = 2).	Counts of HDC, ORX, and MCH; HDC, ORX, MCH, and tyrosine- hydroxylase mRNA expression by qPCR.	HDC neurons and HDC mRNA were unchanged despite ORX deficiency or local neuroinflammation.	Limited ORX-HA mouse availability precluded *a priori* power analysis; the 60-day time point may not capture delayed HDC changes.	No HA recruitment by ORX deficiency or local neuroinflammation in mouse NT1 models.	(30)
	Bastianini et al.	Adult male HDC-KO mice (*n* = 11), DM mice with HDC deficiency and ORX-neuron loss (*n* = 7), and WT controls (*n* = 11).	EEG, EMG, breathing of mice and behavior	Narcolepsy-like phenotypes occurred in DM but not HDC-KO mice.	Non-littermate design and lack of parallel ORX-deficient controls limited assessment of HA effects on ORX-loss phenotypes.	HA deficiency may modulate narcolepsy-related metabolic and respiratory phenotypes.	(31)

NT1, narcolepsy type 1; NC, narcolepsy with cataplexy; NwC, narcolepsy without cataplexy; IH, idiopathic hypersomnia; OSA, obstructive sleep apnea; EDS, excessive daytime sleepiness; Uns EDS, unspecified excessive daytime sleepiness; CSF, cerebrospinal fluid; HA, histamine; t-MH, tele-methylhistamine; ORX, orexin; ORX-A, orexin-A; OX2R, orexin receptor 2; HDC, L-histidine decarboxylase; HDC-KO, L-histidine decarboxylase knockout; ORX-KO, orexin knockout; WT, wild type; DM, double-deficient mice; MCH, melanin-concentrating hormone; DTA, diphtheria toxin A; EEG, electroencephalography; EMG, electromyography; qPCR, quantitative polymerase chain reaction; ORX-HA-2xCD8, transferred twice with autoreactive CD8 T cells; No-T, with no T cells transfer.

Among these factors, differences in the measured markers and sources of evidence may be particularly important. The parameters used across studies are not consistent. HA and t-MH represent different stages of the HA metabolic pathway. The trends in these two markers do not always align. CSF HA levels also cannot be directly equated with central histaminergic activity (28). Central histaminergic function depends not only on HA concentration. It is also influenced by HA synthesis, neuronal firing, axonal projection, synaptic release, metabolic clearance, and receptor sensitivity. This is particularly relevant to signaling mediated by H1R and H3R (22, 33, 34, 40, 55). Therefore, a decrease in CSF HA does not necessarily indicate reduced function of the entire histaminergic system. The absence of significant changes in CSF HA also does not rule out abnormalities in local histaminergic transmission. Similarly, an increase in the number of histaminergic neurons observed in postmortem or animal histological studies cannot be directly interpreted as enhanced histaminergic function. Although some studies have reported increased numbers of HDC-positive neurons in patients with NT1 or in ORX-deficient models, neuronal numbers do not necessarily reflect a corresponding increase in HA release, synaptic output, or receptor-mediated effects (76–78). This type of evidence is better suited to show that ORX deficiency may lead to histological remodeling or compensatory changes in the histaminergic system. It does not directly demonstrate elevated CSF HA levels or an overall increase in histaminergic activity.

Several additional factors may further explain these inconsistent results. First, current studies generally have small sample sizes. NT1 is a rare neurological disorder. Cases with complete CSF collection, comprehensive sleep scale assessments, and detailed medication records are limited (79). Second, there are differences among studies in the age, developmental stage, and disease stage of the included cases. The structural and functional states of histaminergic neural circuits may change depending on the developmental stage and the course of the disease (29, 75). Third, medication history may have influenced the results. Modafinil, sodium oxybate, antidepressants, central nervous system stimulants, and H3R modulators can directly or indirectly affect the histaminergic system and other arousal pathways (80). Fourth, the timing of CSF collection and the patients’ sleep status before sampling varied. HA synthesis and release show distinct circadian rhythms. Their activity levels also fluctuate dynamically across the sleep–wake cycle (81). These factors may affect the interpretation of HA-related measurements, especially when CSF HA or t-MH is used as an indirect marker of central histaminergic function.

Given these limitations, a more conservative conclusion is that CSF HA concentration cannot serve as a stable and reliable diagnostic biomarker for NT1. Findings from CSF HA testing suggest that the histaminergic system may be involved in some disease symptoms, such as EDS and impaired arousal maintenance. However, this marker alone is not sufficient for a definitive assessment of NT1. The histaminergic system may primarily act as a regulatory factor in NT1, rather than as a decisive factor that directly causes the disease.

## 6 The effect of HA on the clinical phenotype of NT1

### 6.1 Excessive daytime sleepiness (EDS)

EDS is the most central, common, and quality-of-life-related symptom in NT1 (82). It mainly presents as a sudden and irresistible need to sleep, often described as a “sleep attack,” and has typical pathological features. Even when patients obtain sufficient sleep at night, their daytime sleepiness is not effectively relieved (83).

Existing mechanistic and clinical evidence confirms that abnormalities in histaminergic system function are closely associated with the development of EDS (27).Pharmacological studies have shown that H1R antagonists lead to drowsiness, whereas H3R inverse agonists increase HA release and effectively relieve sleepiness (84).

### 6.2 Disrupted nighttime sleep (DNS)

DNS is characterized by relatively rapid sleep onset but difficulty maintaining sleep throughout the night, with frequent interruptions of the sleep cycle, repeated brief awakenings, increased sleep-stage transitions, reduced sleep efficiency, and a decreased proportion of SWS (85, 86). These changes can further aggravate EDS (87).

This phenomenon is attributed to ORX deficiency, which causes the body to lose rhythmic regulation of TMN HA neurons and other arousal nuclei, resulting in instability at the sleep–wake boundary (88, 89). Current research has not yet confirmed that histaminergic dysfunction is the primary cause of nighttime sleep fragmentation in NT1.

### 6.3 Cataplexy

Cataplexy is a characteristic symptom of NT1 and is often transiently triggered by strong emotions such as laughter, excitement, surprise, or anger. It manifests as a sudden, brief, and reversible loss of skeletal muscle tone while consciousness is preserved (5). The clinical presentation is highly heterogeneous. Mild cases may present only as ptosis, facial weakness, mandibular drooping, forward head tilt, or knee buckling, whereas severe cases may involve generalized loss of muscle tone and falls. Most episodes last from a few seconds to 1–2 min and then recover spontaneously; patients usually do not fall asleep during an episode (90–92).

It is generally believed that the pathogenesis of cataplexy is related to the substantial loss of orexinergic neurons, after which the muscle-tone inhibitory pathway that normally appears during REM sleep is abnormally activated and intrudes into wakefulness (93). The histaminergic system can indirectly regulate cataplexy, but it is not the key pathogenic factor (94). Insufficient histaminergic signaling may lower the threshold for cataplexy and reduce the ability to resist REM sleep-like muscle atonia during wakefulness (95).

### 6.4 Sleep paralysis and hypnagogic/hypnopompic hallucinations

Sleep paralysis and hypnagogic/hypnopompic hallucinations are common REM sleep-related manifestations of NT1. Sleep paralysis mainly reflects the abnormal persistence or premature onset of REM sleep-related muscle-tone suppression during wakefulness (96, 97), whereas hypnagogic/hypnopompic hallucinations represent abnormal intrusions of dream-like perceptual experiences associated with REM sleep during sleep–wake transitions (98, 99).

Both conditions are primarily associated with ORX deficiency and instability at the sleep–wake boundary, while direct evidence for a specific role of the histaminergic system in these manifestations remains limited.

### 6.5 Cognitive, attentional, and psychiatric symptoms

The histaminergic system is involved in cortical activation, sustained attention, learning and memory, emotional regulation, and motivational behavior; therefore, it may contribute to these non-sleep symptoms (100–102). In NT1, insufficient histaminergic transmission may not only cause daytime sleepiness but also lead to inattention, slowed reaction, and impaired executive function (103). Excessive activation of the histaminergic signaling pathway by H3R inverse agonists may result in side effects such as insomnia, anxiety, hallucinations, and vivid dreams (104, 105).

At present, direct evidence regarding the relationship between the histaminergic system and cognitive or psychiatric symptoms in NT1 remains limited. Most studies have focused on EDS, cataplexy, and nighttime sleep as primary endpoints, with less attention to attention, cognition, and mood.

### 6.6 Pediatric-specific clinical and metabolic phenotypes

In the early stages of the disease, some children with NT1 may exhibit atypical cataplexy, complex motor control disorders, and behavioral and neuropsychiatric symptoms (106–108). However, there is currently a lack of evidence indicating a direct link between these age-specific clinical manifestations and histaminergic abnormalities.

Rapid weight gain, obesity, and metabolic abnormalities are relatively common in children with NT1 (109–111). The histaminergic system is involved in energy balance and metabolic regulation (112, 113). Therefore, alterations in histaminergic function may be associated with body weight and metabolic phenotypes in children with NT1.

## 7 Therapeutic strategies

### 7.1 Targeted therapy drugs

#### 7.1.1 H3R antagonists

H3R antagonists represent the most direct pharmacological approach for targeting the histaminergic system in the treatment of narcolepsy.

Pitolisant is an N-piperidinyl derivative that acts as a high-affinity competitive antagonist and inverse agonist. This drug is currently approved for the symptomatic treatment of narcolepsy and is not classified as a controlled substance (114, 115).

The clinical efficacy of pitolisant is mainly supported by the HARMONY series of studies. HARMONY I, a randomized, double-blind, placebo- and modafinil-controlled study, showed that pitolisant significantly reduced Epworth Sleepiness Scale scores compared with placebo (116). HARMONY-CTP further demonstrated a greater reduction in weekly cataplexy frequency with pitolisant than with placebo (104). Long-term data from HARMONY III suggested sustained efficacy for up to 12 months, although adverse effects such as headache, insomnia, anxiety, depressive symptoms, and nausea were reported (105). Pitolisant may also prolong the QT interval and should be used with caution in patients with known QT prolongation, risk of arrhythmia, impaired liver or kidney function, or concomitant use of other QT-prolonging medications (117).

Samelisant (SUVN-G3031) is a novel and highly selective H3R inverse agonist. It is characterized by good membrane permeability, a high proportion of free drug in plasma, and renal clearance as the primary route of elimination (118, 119).

Unlike pitolisant, samelisant is still an investigational drug. It has not yet been approved for routine clinical treatment of narcolepsy. In terms of clinical development, samelisant has completed a Phase 2 randomized, double-blind, placebo-controlled study. The study included adult patients with narcolepsy, with or without cataplexy. It mainly evaluated the efficacy of samelisant in treating EDS. The study found that samelisant showed some potential benefits in reducing Epworth Sleepiness Scale scores and improving the ability to stay awake. Common adverse reactions included insomnia, abnormal dreams, nausea, and hot flashes. A follow-up Phase 3 study has been registered to further evaluate its efficacy, safety, and tolerability in treating excessive daytime sleepiness associated with narcolepsy (120).

Therefore, samelisant can be considered an H3R-targeted drug candidate with potential for clinical translation. However, the level of evidence supporting samelisant remains lower than that for pitolisant. Its sustained efficacy, long-term safety, and effects on improving cataplexy symptoms require further evaluation (120).

#### 7.1.2 HNMT inhibitors

In addition to H3R inverse agonists, inhibition of HA metabolism is another potential strategy for enhancing central histaminergic signaling. Histamine N-methyltransferase (HNMT) is a key metabolic enzyme involved in the inactivation of HA in the central nervous system. In theory, inhibiting this enzyme can prolong the residence time of HA in the synaptic cleft and enhance histaminergic neurotransmission (121).

Current evidence on HNMT inhibitors mainly comes from basic research and animal studies. Overall, they remain in the early exploratory stages. Animal studies suggest that pharmacological inhibition of HNMT can prolong wakefulness and reduce sudden-fall-like episodes in mouse models of narcolepsy (121, 122). This indicates that this strategy has a degree of mechanistic plausibility.

However, HNMT inhibitors differ from pitolisant, which has already been approved, and samelisant, which is currently in clinical development. HNMT inhibitors still lack evidence from randomized controlled clinical trials in patients with narcolepsy. No clear clinical treatment regimen has been established. Therefore, their translation into clinical practice remains subject to considerable uncertainty.

HA metabolism occurs not only in the central nervous system but also in peripheral tissues. It is also involved in various physiological processes. Therefore, excessive enhancement of histaminergic signaling may lead to insomnia, anxiety, cardiovascular reactions, or other adverse effects (123). Drug development of HNMT inhibitors still needs to address several issues. These include brain targeting, blood–brain barrier permeability, enzyme selectivity, and the therapeutic safety margin (124).

#### 7.1.3 OX2R agonists

TAK-861 is a highly potent, orally available, selective OX2R agonist that directly activates OX2R signaling to compensate for the loss of endogenous ORX signaling in NT1 (125, 126).

Preclinical studies have shown that TAK-861 promotes wakefulness at a low effective dose. It also improves daytime sleepiness and drop-in-place-like behavior in NT1 mice and non-human primate models (125). In terms of clinical development, TAK-861 has completed a Phase 2 randomized, double-blind, placebo-controlled study in patients with NT1. The results suggest that TAK-861 may help improve the ability to stay awake, reduce the severity of daytime sleepiness, and decrease the frequency of cataplexy episodes (126). A subsequent pivotal Phase 3 study further evaluated the efficacy and safety of TAK-861 in patients with NT1. Positive results have been publicly reported. Nevertheless, TAK-861 is still in clinical development and cannot yet be considered an approved standard treatment option. Its efficacy, liver safety, cardiovascular and neuropsychiatric adverse reactions, and potential risks associated with long-term activation of the orexinergic pathway still require further confirmation (127, 128).

Like some other OX2R agonists, TAK-925 and ARN-776 have insufficient brain penetration, which limits their potential as clinical candidates (129). In addition, due to liver safety risks caused by off-target effects, further clinical trials of TAK-994 were halted, preventing the drug from advancing to clinical translation (127, 128, 130).

### 7.2 Comparative interpretation of HA-targeted therapies and OX2R agonists

HA-targeted therapies and OX2R agonists operate at different levels within the ORX–HA arousal regulation network. HA-targeted strategies primarily regulate the arousal network by enhancing histaminergic tone or prolonging HA-mediated neurotransmission (95). In contrast, OX2R agonists act directly on orexin receptor signaling and are therefore mechanistically closer to the core orexin defect in NT1 (10, 128). The two types of strategies also differ in terms of the strength of the evidence and the uncertainty surrounding their implementation. The level of evidence for HA-targeted therapies varies; it includes both drugs already in clinical use and strategies that are still in the clinical trial or preclinical exploration stages (120, 121). OX2R agonists have a clear mechanistic rationale, but their long-term efficacy, safety, and clinical utility require further evaluation (125, 126). Therefore, HA-targeted therapy and OX2R agonists should not be viewed simply as interchangeable treatment options, but rather as two complementary therapeutic strategies.

### 7.3 Comparison with current non-antihistamine treatment strategies

Treatment for narcolepsy currently remains primarily symptomatic. Commonly used non-HA-based pharmacotherapies include oxybate-based medications, wake-promoting agents such as modafinil and solriamfetol, traditional central nervous system stimulants such as methylphenidate, and antidepressants (80, 131). Different drugs mainly target different symptoms (132, 133). Oxybate-class drugs, such as sodium oxybate, low-sodium oxybate, and sustained-release oxybate formulations, are well-established treatment options in current clinical practice (80, 134). These medications are taken at night and primarily improve the continuity of nighttime sleep. They can also reduce EDS and cataplexy. Their advantage lies in relatively robust clinical evidence, and they may be particularly suitable for patients with both cataplexy and nighttime sleep disturbances (135–137). However, their use is also limited by factors such as the need for middle-of-the-night dosing in the case of immediate-release formulations, central nervous system depression, potential abuse, respiratory depression risk, and sodium load, particularly with high-sodium formulations. Newer low-sodium and once-nightly formulations have partly addressed some of these limitations. Therefore, their selection in clinical practice should take into account the patient’s comorbidities, medication adherence, and the need for safety monitoring (138–141).

Antidepressants are mainly used to control REM sleep-related symptoms, such as cataplexy, sleep paralysis, and hypnagogic/hypnopompic hallucinations (132). Their effects are largely associated with enhanced norepinephrine or serotonin transmission and REM sleep inhibition (142). Common side effects include dry mouth, constipation, changes in blood pressure, weight fluctuations, mood changes, and sexual dysfunction (143, 144). Sudden symptom relapse may also occur after discontinuation (143, 145). Therefore, antidepressants are more suitable as adjunctive treatments for managing REM sleep-related symptoms.

Within the current symptom-oriented multidrug treatment framework, HA-related and orexin-targeted strategies further expand the therapeutic targets for NT1 and provide new directions for optimizing future treatment approaches.

## 8 Future research directions and knowledge gaps

Although existing studies suggest that the histaminergic system may be involved in the regulation of NT1 symptoms, its dynamic changes during the disease course remain unclear. Further studies should use more longitudinal designs and enroll patients at different stages of the disease to determine whether histaminergic changes represent a direct response to orexin loss, a compensatory adaptation, or partially independent remodeling within the arousal network, and whether such changes develop progressively over the disease course.

By standardizing the timing of CSF collection, the sleep–wake state before sampling, and the tested parameters, while controlling for medication use, a combined analysis of HA, t-MH, ORX levels, and other arousal-related indicators may help determine whether CSF HA and its metabolites are stable disease-related markers or dynamic indicators influenced by state-dependent changes. In addition, combining these measures with polysomnographic parameters and relevant clinical manifestations may help determine whether the histaminergic system is associated with specific NT1 phenotypes, symptom severity, or treatment response.

The clinical role of HA-related treatment strategies needs to be further clarified. Further work should continue to distinguish between approved drugs, drugs in clinical development, and treatment strategies at the preclinical stage. Long-term follow-up and real-world studies are also needed to evaluate the long-term efficacy, safety, and clinical value of combination therapy. Receptor-specific imaging and patient stratification based on histaminergic responses are also promising research directions. Molecular imaging techniques, such as PET, could be used to assess the distribution and binding of H1R and H3R in key brain regions related to wakefulness. Combined with CSF HA markers and clinical phenotypes, these findings may provide a more direct reflection of the functional state of the histaminergic system in different patient groups. Clinical trials may stratify patients based on baseline HA-related biomarkers, receptor imaging characteristics, or early responses to H3R agents to identify patients who are more likely to benefit from HA-related therapies.

## 9 Conclusion

NT1 is primarily characterized by the selective loss of orexinergic neurons and the resulting disruption of sleep–wake stability. Within this pathophysiological framework, the histaminergic system appears to function as an important component of the broader arousal network rather than as an independent or equivalent driver of the disease. Available anatomical, electrophysiological, and animal evidence suggests a close but functionally asymmetric interaction between the orexinergic and histaminergic systems; however, current evidence remains insufficient to support a simple bidirectional or closed-loop model of regulation between these two systems.

Findings regarding histaminergic alterations in NT1 remain heterogeneous across cerebrospinal fluid studies, postmortem observations, animal models, and pharmacological investigations. These differences suggest that changes in histaminergic signaling may reflect disease-related and state-dependent factors, as well as possible compensatory or developmental processes, rather than a uniform abnormality. Accordingly, the histaminergic system may be more appropriately viewed as a modulatory component of NT1 pathophysiology, potentially contributing to variations in wakefulness maintenance and symptom expression. This interpretation may provide a more balanced framework for interpreting the role of histamine in NT1 and for understanding how histaminergic mechanisms interact with the broader sleep–wake regulatory system.

## References

[1] Ohayon MM Dave S Crawford S Swick TJ Côté ML. Prevalence of narcolepsy in representative samples of the general population of North America, Europe, and South Korea. Psychiatry Res. (2025) 347:116390. doi: 10.1016/j.psychres.2025.116390, 39983282

[2] Scammell TE. Narcolepsy. N Engl J Med. (2015) 373:2654–62. doi: 10.1056/nejmra1500587 26716917

[3] Zhang Z Dauvilliers Y Plazzi G Mayer G Lammers GJ Santamaria J . Idling for decades: a European study on risk factors associated with the delay before a narcolepsy diagnosis. Nat Sci Sleep. (2022) 14:1031–47. doi: 10.2147/NSS.S359980, 35669411 PMC9166906

[4] Nishino S Ripley B Overeem S Lammers GJ Mignot E. Hypocretin (orexin) deficiency in human narcolepsy. Lancet. (2000) 355:39–40. doi: 10.1016/S0140-6736(99)05582-8, 10615891

[5] Sateia MJ. International classification of sleep disorders-third edition: highlights and modifications. Chest. (2014) 146:1387–94. doi: 10.1378/chest.14-0970, 25367475

[6] Morse AM Kim SY Harris S Gow M. Narcolepsy: beyond the classic pentad. CNS Drugs. (2025) 39:9–22. doi: 10.1007/s40263-024-01141-9, 40111737 PMC11950085

[7] Bassetti CLA Adamantidis A Burdakov D Han F Gay S Kallweit U . Narcolepsy - clinical spectrum, aetiopathophysiology, diagnosis and treatment. Nat Rev Neurol. (2019) 15:519–39. doi: 10.1038/s41582-019-0226-9, 31324898

[8] Postiglione E Antelmi E Pizza F Lecendreux M Dauvilliers Y Plazzi G. The clinical spectrum of childhood narcolepsy. Sleep Med Rev. (2018) 38:70–85. doi: 10.1016/j.smrv.2017.04.003, 28666745

[9] Rocca FL Pizza F Ricci E Plazzi G. Narcolepsy during childhood: an update. Neuropediatrics. (2015) 46:181–98. doi: 10.1055/s-0035-1550152, 25961600

[10] Mahoney CE Cogswell A Koralnik IJ Scammell TE. The neurobiological basis of narcolepsy. Nat Rev Neurosci. (2019) 20:83–93. doi: 10.1038/s41583-018-0097-x, 30546103 PMC6492289

[11] Thannickal TC Moore RY Nienhuis R Ramanathan L Gulyani S Aldrich M . Reduced number of hypocretin neurons in human narcolepsy. Neuron. (2000) 27:469–74. doi: 10.1016/s0896-6273(00)00058-1, 11055430 PMC8760623

[12] Peyron C Faraco J Rogers W Ripley B Overeem S Charnay Y . A mutation in a case of early onset narcolepsy and a generalized absence of hypocretin peptides in human narcoleptic brains. Nat Med. (2000) 6:991–7. doi: 10.1038/79690, 10973318

[13] Lin L Faraco J Li R Kadotani H Rogers W Lin X . The sleep disorder canine narcolepsy is caused by a mutation in the hypocretin (orexin) receptor 2 gene. Cell. (1999) 98:365–76. doi: 10.1016/s0092-8674(00)81965-0, 10458611

[14] Chemelli RM Willie JT Sinton CM Elmquist JK Scammell T Lee C . Narcolepsy in orexin knockout mice: molecular genetics of sleep regulation. Cell. (1999) 98:437–51. doi: 10.1016/s0092-8674(00)81973-x, 10481909

[15] Hara J Beuckmann CT Nambu T Willie JT Chemelli RM Sinton CM . Genetic ablation of orexin neurons in mice results in narcolepsy, hypophagia, and obesity. Neuron. (2001) 30:345–54. doi: 10.1016/S0896-6273(01)00293-8, 11394998

[16] Bonvalet M Ollila HM Ambati A Mignot E. Autoimmunity in narcolepsy. Curr Opin Pulm Med. (2017) 23:522–9. doi: 10.1097/MCP.0000000000000426, 28991006 PMC5773260

[17] Buonocore SM van der Most RG. Narcolepsy and H1N1 influenza immunology a decade later: what have we learned? Front Immunol. (2022) 13:902840. doi: 10.3389/fimmu.2022.902840, 36311717 PMC9601309

[18] Latorre D Kallweit U Armentani E Foglierini M Mele F Cassotta A . T cells in patients with narcolepsy target self-antigens of hypocretin neurons. Nature. (2018) 562:63–8. doi: 10.1038/s41586-018-0540-1, 30232458

[19] Date Y Ueta Y Yamashita H Yamaguchi H Matsukura S Kangawa K . Orexins, orexigenic hypothalamic peptides, interact with autonomic, neuroendocrine and neuroregulatory systems. Proc Natl Acad Sci USA. (1999) 96:748–53. doi: 10.1073/pnas.96.2.748, 9892705 PMC15208

[20] Peyron C Tighe DK van den Pol AN de Lecea L Heller HC Sutcliffe JG . Neurons containing hypocretin (orexin) project to multiple neuronal systems. J Neurosci. (1998) 18:9996–10015. doi: 10.1523/JNEUROSCI.18-23-09996.1998, 9822755 PMC6793310

[21] Sakurai T. The neural circuit of orexin (hypocretin): maintaining sleep and wakefulness. Nat Rev Neurosci. (2007) 8:171–81. doi: 10.1038/nrn2092, 17299454

[22] Yoshikawa T Nakamura T Yanai K. Histaminergic neurons in the tuberomammillary nucleus as a control Centre for wakefulness. Br J Pharmacol. (2021) 178:750–69. doi: 10.1111/bph.15220, 32744724

[23] Fujita A Bonnavion P Wilson MH Mickelsen LE Bloit J de Lecea L . Hypothalamic Tuberomammillary nucleus neurons: electrophysiological diversity and essential role in arousal stability. J Neurosci. (2017) 37:9574–92. doi: 10.1523/JNEUROSCI.0580-17.2017, 28874450 PMC5618271

[24] Parmentier R Ohtsu H Djebbara-Hannas Z Valatx JL Watanabe T Lin JS. Anatomical, physiological, and pharmacological characteristics of histidine decarboxylase knock-out mice: evidence for the role of brain histamine in behavioral and sleep-wake control. J Neurosci. (2002) 22:7695–711. doi: 10.1523/JNEUROSCI.22-17-07695.2002, 12196593 PMC6757981

[25] Nishino S Sakurai E Nevsimalova S Yoshida Y Watanabe T Yanai K . Decreased CSF histamine in narcolepsy with and without low CSF hypocretin-1 in comparison to healthy controls. Sleep. (2009) 32:175–80. doi: 10.1093/sleep/32.2.175, 19238804 PMC2635581

[26] Kanbayashi T Kodama T Kondo H Satoh S Inoue Y Chiba S . CSF histamine contents in narcolepsy, idiopathic hypersomnia and obstructive sleep apnea syndrome. Sleep. (2009) 32:181–7. doi: 10.1093/sleep/32.2.181, 19238805 PMC2635582

[27] Bassetti CL Baumann CR Dauvilliers Y Croyal M Robert P Schwartz JC. Cerebrospinal fluid histamine levels are decreased in patients with narcolepsy and excessive daytime sleepiness of other origin. J Sleep Res. (2010) 19:620–3. doi: 10.1111/j.1365-2869.2010.00819.x, 20846244

[28] Croyal M Dauvilliers Y Labeeuw O Capet M Schwartz JC Robert P. Histamine and tele-methylhistamine quantification in cerebrospinal fluid from narcoleptic subjects by liquid chromatography tandem mass spectrometry with precolumn derivatization. Anal Biochem. (2011) 409:28–36. doi: 10.1016/j.ab.2010.09.045, 20932951

[29] Lopez R Barateau L Evangelista E Chenini S Robert P Jaussent I . Temporal changes in the cerebrospinal fluid level of Hypocretin-1 and histamine in narcolepsy. Sleep. (2017) 40.zsw010. doi: 10.1093/sleep/zsw010, 28364477 PMC5806580

[30] Melzi S Morel AL Scoté-Blachon C Liblau R Dauvilliers Y Peyron C. Histamine in murine narcolepsy: what do genetic and immune models tell us? Brain Pathol. (2022) 32:e13027. doi: 10.1111/bpa.13027, 34672414 PMC8877734

[31] Bastianini S Silvani A Berteotti C Lo Martire V Cohen G Ohtsu H . Histamine transmission modulates the phenotype of murine narcolepsy caused by orexin neuron deficiency. PLoS One. (2015) 10:e0140520. doi: 10.1371/journal.pone.0140520, 26474479 PMC4608736

[32] Lin JS Kitahama K Fort P Panula P Denney RM Jouvet M. Histaminergic system in the cat hypothalamus with reference to type B monoamine oxidase. J Comp Neurol. (1993) 330:405–20. doi: 10.1002/cne.903300309, 7682224

[33] Haas HL Sergeeva OA Selbach O. Histamine in the nervous system. Physiol Rev. (2008) 88:1183–241. doi: 10.1152/physrev.00043.2007, 18626069

[34] Thakkar MM. Histamine in the regulation of wakefulness. Sleep Med Rev. (2011) 15:65–74. doi: 10.1016/j.smrv.2010.06.004, 20851648 PMC3016451

[35] Lee MG Hassani OK Jones BE. Discharge of identified orexin/hypocretin neurons across the sleep-waking cycle. J Neurosci. (2005) 25:6716–20. doi: 10.1523/JNEUROSCI.1887-05.2005, 16014733 PMC6725432

[36] Jørgensen EA Knigge U Warberg J Kjaer A. Histamine and the regulation of body weight. Neuroendocrinology. (2007) 86:210–4. doi: 10.1159/000108341, 17848791

[37] Branco A Yoshikawa FSY Pietrobon AJ Sato MN. Role of histamine in modulating the immune response and inflammation. Mediat Inflamm. (2018) 2018:1–10. doi: 10.1155/2018/9524075, 30224900 PMC6129797

[38] Panula P Yang HY Costa E. Histamine-containing neurons in the rat hypothalamus. Proc Natl Acad Sci USA. (1984) 81:2572–6. doi: 10.1073/pnas.81.8.2572, 6371818 PMC345105

[39] Haas H Panula P. The role of histamine and the tuberomamillary nucleus in the nervous system. Nat Rev Neurosci. (2003) 4:121–30. doi: 10.1038/nrn1034, 12563283

[40] Krystal AD Richelson E Roth T. Review of the histamine system and the clinical effects of H1 antagonists: basis for a new model for understanding the effects of insomnia medications. Sleep Med Rev. (2013) 17:263–72. doi: 10.1016/j.smrv.2012.08.001, 23357028

[41] Takahashi K Lin JS Sakai K. Neuronal activity of histaminergic tuberomammillary neurons during wake-sleep states in the mouse. J Neurosci. (2006) 26:10292–8. doi: 10.1523/JNEUROSCI.2341-06.2006, 17021184 PMC6674640

[42] Tuomisto L Lozeva V Valjakka A Lecklin A. Modifying effects of histamine on circadian rhythms and neuronal excitability. Behav Brain Res. (2001) 124:129–35. doi: 10.1016/S0166-4328(01)00222-4, 11640965

[43] Jewett RE. Effects of promethazine on sleep stages in the cat. Exp Neurol. (1968) 21:368–82. doi: 10.1016/0014-4886(68)90048-4, 5673651

[44] Wauquier A Van den Broeck WA Awouters F Janssen PA. A comparison between astemizole and other antihistamines on sleep-wakefulness cycles in dogs. Neuropharmacology. (1981) 20:853–9. doi: 10.1016/0028-3908(81)90078-2, 6117034

[45] Risberg AM Risberg J Ingvar DH. Effects of promethazine on nocturnal sleep in normal man. Psychopharmacologia. (1975) 43:279–84. doi: 10.1007/BF00429264, 171695

[46] Kalivas PW. Histamine-induced arousal in the conscious and pentobarbital-pretreated rat. J Pharmacol Exp Ther. (1982) 222:37–42. doi: 10.1016/S0022-3565(25)33149-6, 7086707

[47] Huang ZL Mochizuki T Qu WM Hong ZY Watanabe T Urade Y . Altered sleep-wake characteristics and lack of arousal response to H3 receptor antagonist in histamine H1 receptor knockout mice. Proc Natl Acad Sci USA. (2006) 103:4687–92. doi: 10.1073/pnas.0600451103, 16537376 PMC1450232

[48] Ishiguro T Iwase M Kanamaru M Izumizaki M Ohshima Y Homma I. Impaired ventilation and metabolism response to hypoxia in histamine H1 receptor-knockout mice. Respir Physiol Neurobiol. (2006) 154:331–41. doi: 10.1016/j.resp.2006.01.017, 16540381

[49] Parmentier R Zhao Y Perier M Akaoka H Lintunen M Hou Y . Role of histamine H1-receptor on behavioral states and wake maintenance during deficiency of a brain activating system: a study using a knockout mouse model. Neuropharmacology. (2016) 106:20–34. doi: 10.1016/j.neuropharm.2015.12.014, 26723880

[50] Lieberman P. Histamine, antihistamines, and the central nervous system. Allergy Asthma Proc. (2009) 30:482–6. doi: 10.2500/aap.2009.30.3264, 19843401

[51] Van Ruitenbeek P Vermeeren A Riedel WJ. Cognitive domains affected by histamine H(1)-antagonism in humans: a literature review. Brain Res Rev. (2010) 64:263–82. doi: 10.1016/j.brainresrev.2010.04.008, 20685608

[52] Yanai K Zhang D Tashiro M Yoshikawa T Naganuma F Harada R . Positron emission tomography evaluation of sedative properties of antihistamines. Expert Opin Drug Saf. (2011) 10:613–22. doi: 10.1517/14740338.2011.562889, 21521134

[53] Panula P Chazot PL Cowart M Gutzmer R Leurs R Liu WL . International union of basic and clinical pharmacology. XCVIII. Histamine receptors. Pharmacol Rev. (2015) 67:601–55. doi: 10.1124/pr.114.010249, 26084539 PMC4485016

[54] Pillot C Heron A Cochois V Tardivel-Lacombe J Ligneau X Schwartz JC . A detailed mapping of the histamine H(3) receptor and its gene transcripts in rat brain. Neuroscience. (2002) 114:173–93. doi: 10.1016/S0306-4522(02)00135-5, 12207964

[55] Arrang JM Garbarg M Schwartz JC. Autoinhibition of histamine synthesis mediated by presynaptic H3-receptors. Neuroscience. (1987) 23:149–57. doi: 10.1016/0306-4522(87)90279-X, 2446202

[56] Nieto-Alamilla G Márquez-Gómez R García-Gálvez AM Morales-Figueroa GE Arias-Montaño JA. The histamine H3 receptor: structure, pharmacology, and function. Mol Pharmacol. (2016) 90:649–73. doi: 10.1124/mol.116.104752, 27563055

[57] Lin JS Anaclet C Sergeeva OA Haas HL. The waking brain: an update. Cell Mol Life Sci. (2011) 68:2499–512. doi: 10.1007/s00018-011-0631-8, 21318261 PMC3134769

[58] Lin JS Sakai K Vanni-Mercier G Arrang JM Garbarg M Schwartz JC . Involvement of histaminergic neurons in arousal mechanisms demonstrated with H3-receptor ligands in the cat. Brain Res. (1990) 523:325–30. doi: 10.1016/0006-8993(90)91508-E, 2169324

[59] Monti JM Jantos H Boussard M Altier H Orellana C Olivera S. Effects of selective activation or blockade of the histamine H3 receptor on sleep and wakefulness. Eur J Pharmacol. (1991) 205:283–7. doi: 10.1016/0014-2999(91)90911-9, 1667912

[60] Stocking EM Letavic MA. Histamine H3 antagonists as wake-promoting and pro-cognitive agents. Curr Top Med Chem. (2008) 8:988–1002. doi: 10.2174/156802608784936728, 18673168

[61] Giannoni P Medhurst AD Passani MB Giovannini MG Ballini C Corte LD . Regional differential effects of the novel histamine H3 receptor antagonist 6-[(3-cyclobutyl-2,3,4,5-tetrahydro-1H-3-benzazepin-7-yl)oxy]-N-methyl-3-pyridinecarboxamide hydrochloride (GSK189254) on histamine release in the central nervous system of freely moving rats. J Pharmacol Exp Ther. (2010) 332:164–72. doi: 10.1124/jpet.109.158444, 19815811 PMC2802467

[62] Medhurst AD Atkins AR Beresford IJ Brackenborough K Briggs MA Calver AR . GSK189254, a novel H3 receptor antagonist that binds to histamine H3 receptors in Alzheimer's disease brain and improves cognitive performance in preclinical models. J Pharmacol Exp Ther. (2007) 321:1032–45. doi: 10.1124/jpet.107.120311, 17327487

[63] Harwell V Fasinu PS. Pitolisant and other histamine-3 receptor antagonists-an update on therapeutic potentials and clinical prospects. Medicines Basel. (2020) 7.55 doi: 10.3390/medicines7090055, 32882898 PMC7554886

[64] Guevarra JT Hiensch R Varga AW Rapoport DM. Pitolisant to treat excessive daytime sleepiness and cataplexy in adults with narcolepsy: rationale and clinical utility. Nat Sci Sleep. (2020) 12:709–19. doi: 10.2147/NSS.S264140, 33117007 PMC7567539

[65] Yamanaka A Tsujino N Funahashi H Honda K Guan JL Wang QP . Orexins activate histaminergic neurons via the orexin 2 receptor. Biochem Biophys Res Commun. (2002) 290:1237–45. doi: 10.1006/bbrc.2001.6318, 11811995

[66] Eriksson KS Sergeeva O Brown RE Haas HL. Orexin/hypocretin excites the histaminergic neurons of the tuberomammillary nucleus. J Neurosci. (2001) 21:9273–9. doi: 10.1523/JNEUROSCI.21-23-09273.2001, 11717361 PMC6763926

[67] Schöne C Cao ZF Apergis-Schoute J Adamantidis A Sakurai T Burdakov D. Optogenetic probing of fast glutamatergic transmission from hypocretin/orexin to histamine neurons in situ. J Neurosci. (2012) 32:12437–43. doi: 10.1523/JNEUROSCI.0706-12.2012, 22956835 PMC6621251

[68] Huang ZL Qu WM Li WD Mochizuki T Eguchi N Watanabe T . Arousal effect of orexin a depends on activation of the histaminergic system. Proc Natl Acad Sci USA. (2001) 98:9965–70. doi: 10.1073/pnas.181330998, 11493714 PMC55561

[69] Cheng L Liu J Chen Z. The histaminergic system in neuropsychiatric disorders. Biomolecules. (2021) 11:1345 doi: 10.3390/biom11091345, 34572558 PMC8467868

[70] Ishikawa T Kurimoto E Joyal AA Koike T Kimura H Scammell TE. An orexin agonist promotes wakefulness and inhibits cataplexy through distinct brain regions. Curr Biol. (2025) 35:2088–99.e4. doi: 10.1016/j.cub.2025.03.040, 40233754

[71] Sundvik M Kudo H Toivonen P Rozov S Chen YC Panula P. The histaminergic system regulates wakefulness and orexin/hypocretin neuron development via histamine receptor H1 in zebrafish. FASEB J. (2011) 25:4338–47. doi: 10.1096/fj.11-188268, 21885652

[72] Giannoni P Passani MB Nosi D Chazot PL Shenton FC Medhurst AD . Heterogeneity of histaminergic neurons in the tuberomammillary nucleus of the rat. Eur J Neurosci. (2009) 29:2363–74. doi: 10.1111/j.1460-9568.2009.06765.x, 19490084

[73] Haas HL Lin JS. Waking with the hypothalamus. Pflugers Arch. (2012) 463:31–42. doi: 10.1007/s00424-011-0996-4, 21796339

[74] Dauvilliers Y Delallée N Jaussent I Scholz S Bayard S Croyal M . Normal cerebrospinal fluid histamine and tele-methylhistamine levels in hypersomnia conditions. Sleep. (2012) 35:1359–66. doi: 10.5665/sleep.2114, 23024434 PMC3443762

[75] Franco P Dauvilliers Y Inocente CO Guyon A Villanueva C Raverot V . Impaired histaminergic neurotransmission in children with narcolepsy type 1. CNS Neurosci Ther. (2019) 25:386–95. doi: 10.1111/cns.13057, 30225986 PMC6488909

[76] John J Thannickal TC McGregor R Ramanathan L Ohtsu H Nishino S . Greatly increased numbers of histamine cells in human narcolepsy with cataplexy. Ann Neurol. (2013) 74:786–93. doi: 10.1002/ana.23968, 23821583 PMC8211429

[77] Valko PO Gavrilov YV Yamamoto M Reddy H Haybaeck J Mignot E . Increase of histaminergic tuberomammillary neurons in narcolepsy. Ann Neurol. (2013) 74:794–804. doi: 10.1002/ana.24019, 24006291

[78] Berteotti C Lo Martire V Alvente S Bastianini S Bombardi C Matteoli G . Orexin/Hypocretin and histamine cross-talk on hypothalamic neuron counts in mice. Front Neurosci. (2021) 15:660518. doi: 10.3389/fnins.2021.660518, 34093114 PMC8173058

[79] Ohayon MM Duhoux S Grieco J Côté ML. Prevalence and incidence of narcolepsy symptoms in the US general population. Sleep Med X. (2023) 6:100095. doi: 10.1016/j.sleepx.2023.100095, 38149177 PMC10749896

[80] Maski K Trotti LM Kotagal S Robert Auger R Rowley JA Hashmi SD . Treatment of central disorders of hypersomnolence: an American Academy of sleep medicine clinical practice guideline. J Clin Sleep Med. (2021) 17:1881–93. doi: 10.5664/jcsm.9328, 34743789 PMC8636351

[81] Zeitzer JM Kodama T Buckmaster CL Honda Y Lyons DM Nishino S . Time-course of cerebrospinal fluid histamine in the wake-consolidated squirrel monkey. J Sleep Res. (2012) 21:189–94. doi: 10.1111/j.1365-2869.2011.00957.x, 21910776 PMC3237761

[82] Galušková K Nevšímalová S Příhodová I Dostálová S Mazouchová A Šonka K. Quality of life and its associates in narcolepsy 1 and 2 types and idiopathic hypersomnia. Sleep Med. (2025) 129:31–9. doi: 10.1016/j.sleep.2025.02.019, 39978241

[83] Zeman A Britton T Douglas N Hansen A Hicks J Howard R . Narcolepsy and excessive daytime sleepiness. BMJ. (2004) 329:724–8. doi: 10.1136/bmj.329.7468.724, 15388615 PMC518900

[84] Scammell TE Jackson AC Franks NP Wisden W Dauvilliers Y. Histamine: neural circuits and new medications. Sleep. (2019) 42.zsy183. doi: 10.1093/sleep/zsy183, 30239935 PMC6335869

[85] Maski K Mignot E Plazzi G Dauvilliers Y. Disrupted nighttime sleep and sleep instability in narcolepsy. J Clin Sleep Med. (2022) 18:289–304. doi: 10.5664/jcsm.9638, 34463249 PMC8807887

[86] Zhang Y Ren R Yang L Zhang H Shi Y Sanford LD . Polysomnographic nighttime features of narcolepsy: a systematic review and meta-analysis. Sleep Med Rev. (2021) 58:101488. doi: 10.1016/j.smrv.2021.101488, 33934047

[87] Khatami R Landolt HP Achermann P Rétey JV Werth E Mathis J . Insufficient non-REM sleep intensity in narcolepsy-cataplexy. Sleep. (2007) 30:980–9. doi: 10.1093/sleep/30.8.980, 17702267 PMC1978383

[88] Sorensen GL Knudsen S Jennum P. Sleep transitions in hypocretin-deficient narcolepsy. Sleep. (2013) 36:1173–7. doi: 10.5665/sleep.2880, 23904677 PMC3700714

[89] Alakuijala A Sarkanen T Partinen M. Hypocretin-1 levels associate with fragmented sleep in patients with narcolepsy type 1. Sleep. (2016) 39:1047–50. doi: 10.5665/sleep.5750, 26856902 PMC4835302

[90] Pizza F Antelmi E Vandi S Meletti S Erro R Baumann CR . The distinguishing motor features of cataplexy: a study from video-recorded attacks. Sleep. (2018) 41:zsy026 doi: 10.1093/sleep/zsy026, 29425380

[91] Dauvilliers Y Siegel JM Lopez R Torontali ZA Peever JH. Cataplexy—clinical aspects, pathophysiology and management strategy. Nat Rev Neurol. (2014) 10:386–95. doi: 10.1038/nrneurol.2014.97, 24890646 PMC8788644

[92] John J Wu MF Boehmer LN Siegel JM. Cataplexy-active neurons in the hypothalamus: implications for the role of histamine in sleep and waking behavior. Neuron. (2004) 42:619–34. doi: 10.1016/S0896-6273(04)00247-8, 15157423 PMC8765806

[93] Thorpy MJ Siegel JM Dauvilliers Y. REM sleep in narcolepsy. Sleep Med Rev. (2024) 77:101976. doi: 10.1016/j.smrv.2024.101976, 39186901

[94] Shan L Dauvilliers Y Siegel JM. Interactions of the histamine and hypocretin systems in CNS disorders. Nat Rev Neurol. (2015) 11:401–13. doi: 10.1038/nrneurol.2015.99, 26100750 PMC8744538

[95] Lin JS Dauvilliers Y Arnulf I Bastuji H Anaclet C Parmentier R . An inverse agonist of the histamine H(3) receptor improves wakefulness in narcolepsy: studies in orexin−/− mice and patients. Neurobiol Dis. (2008) 30:74–83. doi: 10.1016/j.nbd.2007.12.003, 18295497

[96] Stefani A Högl B. Nightmare disorder and isolated sleep paralysis. Neurotherapeutics. (2021) 18:100–6. doi: 10.1007/s13311-020-00966-8, 33230689 PMC8116464

[97] Nishino S Okuro M Kotorii N Anegawa E Ishimaru Y Matsumura M . Hypocretin/orexin and narcolepsy: new basic and clinical insights. Acta Physiol (Oxf). (2010) 198:209–22. doi: 10.1111/j.1748-1716.2009.02012.x, 19555382 PMC2860658

[98] Fortuyn HA Lappenschaar GA Nienhuis FJ Furer JW Hodiamont PP Rijnders CA . Psychotic symptoms in narcolepsy: phenomenology and a comparison with schizophrenia. Gen Hosp Psychiatry. (2009) 31:146–54. doi: 10.1016/j.genhosppsych.2008.12.002, 19269535

[99] Waters F Blom JD Dang-Vu TT Cheyne AJ Alderson-Day B Woodruff P . What is the link between hallucinations, dreams, and hypnagogic-hypnopompic experiences? Schizophr Bull. (2016) 42:1098–109. doi: 10.1093/schbul/sbw076, 27358492 PMC4988750

[100] Passani MB Bacciottini L Mannaioni PF Blandina P. Central histaminergic system and cognition. Neurosci Biobehav Rev. (2000) 24:107–13. doi: 10.1016/S0149-7634(99)00053-6, 10654665

[101] Blandina P Munari L Provensi G Passani MB. Histamine neurons in the tuberomamillary nucleus: a whole center or distinct subpopulations? Front Syst Neurosci. (2012) 6:33. doi: 10.3389/fnsys.2012.00033, 22586376 PMC3343474

[102] Torrealba F Riveros ME Contreras M Valdes JL. Histamine and motivation. Front Syst Neurosci. (2012) 6:51. doi: 10.3389/fnsys.2012.00051, 22783171 PMC3389384

[103] Harel BT Gattuso JJ Latzman RD Maruff P Scammell TE Plazzi G. The nature and magnitude of cognitive impairment in narcolepsy type 1, narcolepsy type 2, and idiopathic hypersomnia: a meta-analysis. Sleep Adv. (2024) 5:zpae043. doi: 10.1093/sleepadvances/zpae043, 39036743 PMC11258808

[104] Szakacs Z Dauvilliers Y Mikhaylov V Poverennova I Krylov S Jankovic S . Safety and efficacy of pitolisant on cataplexy in patients with narcolepsy: a randomised, double-blind, placebo-controlled trial. Lancet Neurol. (2017) 16:200–7. doi: 10.1016/S1474-4422(16)30333-7, 28129985

[105] Dauvilliers Y Arnulf I Szakacs Z Leu-Semenescu S Lecomte I Scart-Gres C . Long-term use of pitolisant to treat patients with narcolepsy: Harmony III study. Sleep. (2019) 42:zsz174. doi: 10.1093/sleep/zsz174, 31529094 PMC6802569

[106] Plazzi G Pizza F Palaia V Franceschini C Poli F Moghadam KK . Complex movement disorders at disease onset in childhood narcolepsy with cataplexy. Brain. (2011) 134:3477–89. doi: 10.1093/brain/awr244, 21930661 PMC3235554

[107] Antelmi E Pizza F Vandi S Neccia G Ferri R Bruni O . The spectrum of REM sleep-related episodes in children with type 1 narcolepsy. Brain. (2017) 140:1669–79. doi: 10.1093/brain/awx096, 28472332

[108] Veneruso M Pizza F Finotti E Amore G Vandi S Filardi M . Child neurology: a case series of heterogeneous neuropsychiatric symptoms and outcome in very early-onset narcolepsy type 1. Neurology. (2022) 98:984–9. doi: 10.1212/WNL.0000000000200666, 35387850

[109] Zhang M Thieux M Inocente CO Vieux N Arvis L Villanueva C . Characterization of rapid weight gain phenotype in children with narcolepsy. CNS Neurosci Ther. (2022) 28:829–41. doi: 10.1111/cns.13811, 35212159 PMC9062543

[110] Zhang M Thieux M Arvis L Lin JS Guyon A Plancoulaine S . Metabolic disturbances in children with narcolepsy: a retrospective study. Sleep. (2023) 46:zsad076 doi: 10.1093/sleep/zsad076, 36971181

[111] Casale S Assirelli V Pizza F Balsamo A Gennari M Pession A . Auxological and endocrine findings in narcolepsy type 1: seventeen-year follow-up from a pediatric endocrinology center. Front Endocrinol (Lausanne). (2023) 14:1037398. doi: 10.3389/fendo.2023.1037398, 37396177 PMC10311432

[112] Khouma A Moeini MM Plamondon J Richard D Caron A Michael NJ. Histaminergic regulation of food intake. Front Endocrinol (Lausanne). (2023) 14:1202089. doi: 10.3389/fendo.2023.1202089, 37448468 PMC10338010

[113] Masaki T Yoshimatsu H. Neuronal histamine and its receptors: implication of the pharmacological treatment of obesity. Curr Med Chem. (2010) 17:4587–92. doi: 10.2174/092986710794182944, 21062248

[114] Schwartz JC. The histamine H3 receptor: from discovery to clinical trials with pitolisant. Br J Pharmacol. (2011) 163:713–21. doi: 10.1111/j.1476-5381.2011.01286.x, 21615387 PMC3111674

[115] Lamb YN. Pitolisant: a review in narcolepsy with or without cataplexy. CNS Drugs. (2020) 34:207–18. doi: 10.1007/s40263-020-00703-x, 31997137

[116] Dauvilliers Y Bassetti C Lammers GJ Arnulf I Mayer G Rodenbeck A . Pitolisant versus placebo or modafinil in patients with narcolepsy: a double-blind, randomised trial. Lancet Neurol. (2013) 12:1068–75. doi: 10.1016/S1474-4422(13)70225-4, 24107292

[117] Thorpy MJ. Recently approved and upcoming treatments for narcolepsy. CNS Drugs. (2020) 34:9–27. doi: 10.1007/s40263-019-00689-1, 31953791 PMC6982634

[118] Nirogi R Benade V Daripelli S Subramanian R Kamuju V Bhyrapuneni G . Samelisant (SUVN-G3031), a potent, selective and orally active histamine H3 receptor inverse agonist for the potential treatment of narcolepsy: pharmacological and neurochemical characterisation. Psychopharmacology. (2021) 238:1495–511. doi: 10.1007/s00213-021-05779-x, 33550481

[119] Nirogi R Bhyrapuneni G Muddana NR Manoharan A Shinde AK Mohammed AR . Absorption, distribution, metabolism, excretion (ADME), drug-drug interaction potential and prediction of human pharmacokinetics of SUVN-G3031, a novel histamine 3 receptor (H(3)R) inverse agonist in clinical development for the treatment of narcolepsy. Eur J Pharm Sci. (2020) 152:105425. doi: 10.1016/j.ejps.2020.105425, 32534194

[120] Nirogi R Shinde A Goyal VK Ravula J Benade V Jetta S . Samelisant (SUVN-G3031), a histamine 3 receptor inverse agonist: results from the phase 2 double-blind randomized placebo-controlled study for the treatment of excessive daytime sleepiness in adult patients with narcolepsy. Sleep Med. (2024) 124:618–26. doi: 10.1016/j.sleep.2024.10.037, 39504585

[121] Naganuma F Girgin B Agu ABS Hirano K Nakamura T Yanai K . Pharmacological inhibition of histamine N-methyltransferase extends wakefulness and suppresses cataplexy in a mouse model of narcolepsy. Sleep. (2025) 48:zsae244. doi: 10.1093/sleep/zsae244, 39441998 PMC12477117

[122] Naganuma F Nakamura T Yoshikawa T Iida T Miura Y Kárpáti A . Histamine N-methyltransferase regulates aggression and the sleep-wake cycle. Sci Rep. (2017) 7:15899. doi: 10.1038/s41598-017-16019-8, 29162912 PMC5698467

[123] Jochem J. Cardiac and regional haemodynamic effects of histamine N-methyltransferase inhibitor metoprine in haemorrhage-shocked rats. Inflamm Res. (2004) 53:316–23. doi: 10.1007/s00011-004-1268-y, 15241567

[124] Yoshikawa T Nakamura T Yanai K. Histamine N-Methyltransferase in the brain. Int J Mol Sci. (2019) 20:737. doi: 10.3390/ijms20030737, 30744146 PMC6386932

[125] Mitsukawa K Terada M Yamada R Monjo T Hiyoshi T Nakakariya M . TAK-861, a potent, orally available orexin receptor 2-selective agonist, produces wakefulness in monkeys and improves narcolepsy-like phenotypes in mouse models. Sci Rep. (2024) 14:20838. doi: 10.1038/s41598-024-70594-1, 39242684 PMC11379823

[126] Dauvilliers Y Plazzi G Mignot E Lammers GJ Del Río Villegas R Khatami R . Oveporexton, an oral orexin receptor 2-selective agonist, in narcolepsy type 1. N Engl J Med. (2025) 392:1905–16. doi: 10.1056/nejmoa2405847 40367374

[127] Shinozawa T Miyamoto K Baker KS Faber SC Flores R Uetrecht J . TAK-994 mechanistic investigation into drug-induced liver injury. Toxicol Sci. (2025) 204:143–53. doi: 10.1093/toxsci/kfaf003, 39786842 PMC11939078

[128] Dauvilliers Y Mignot E Del Río Villegas R Du Y Hanson E Inoue Y . Oral orexin receptor 2 agonist in narcolepsy type 1. N Engl J Med. (2023) 389:309–21. doi: 10.1056/nejmoa2301940, 37494485

[129] Sun Y Ranjan A Tisdale R Ma SC Park S Haire M . Evaluation of the efficacy of the hypocretin/orexin receptor agonists TAK-925 and ARN-776 in narcoleptic orexin/tTA; TetO-DTA mice. J Sleep Res. (2023) 32:e13839. doi: 10.1111/jsr.13839. PMC10356740 36808670

[130] Ishikawa T Hara H Kawano A Tohyama K Kajita Y Miyanohana Y . TAK-994, a novel orally available brain-penetrant orexin 2 receptor-selective agonist, suppresses fragmentation of wakefulness and cataplexy-like episodes in mouse models of narcolepsy. J Pharmacol Exp Ther. (2023) 385:193–204. doi: 10.1124/jpet.122.001449, 37001988

[131] Zhou X Chen J Xu B Chen L. Evaluation of pitolisant, sodium oxybate, solriamfetol, and modafinil for the management of narcolepsy: a retrospective analysis of the FAERS database. Front Pharmacol. (2024) 15:1415918. doi: 10.3389/fphar.2024.1415918, 39588154 PMC11586220

[132] Bassetti CLA Kallweit U Vignatelli L Plazzi G Lecendreux M Baldin E . European guideline and expert statements on the management of narcolepsy in adults and children. J Sleep Res. (2021) 30:e13387. doi: 10.1111/jsr.13387, 34173288

[133] Thorpy MJ Dauvilliers Y. Clinical and practical considerations in the pharmacologic management of narcolepsy. Sleep Med. (2015) 16:9–18. doi: 10.1016/j.sleep.2014.10.002, 25458251

[134] Roth T. Therapeutic use of γ-Hydroxybutyrate: history and clinical utility of Oxybates and considerations of once- and twice-nightly dosing in narcolepsy. CNS Drugs. (2025) 39:37–51. doi: 10.1007/s40263-024-01150-8, 40111735 PMC11950157

[135] Black J Pardi D Hornfeldt CS Inhaber N. The nightly use of sodium oxybate is associated with a reduction in nocturnal sleep disruption: a double-blind, placebo-controlled study in patients with narcolepsy. J Clin Sleep Med. (2010) 6:596–602. doi: 10.5664/jcsm.27994 PMC3014247 21206549

[136] Roth T Dauvilliers Y Guinta D Alvarez-Horine S Dynin E Black J. Effect of sodium oxybate on disrupted nighttime sleep in patients with narcolepsy. J Sleep Res. (2017) 26:407–14. doi: 10.1111/jsr.12468, 27807903

[137] Roth T Dauvilliers Y Thorpy MJ Kushida C Corser BC Bogan R . Effect of FT218, a once-nightly sodium Oxybate formulation, on disrupted nighttime sleep in patients with narcolepsy: results from the randomized phase III REST-ON trial. CNS Drugs. (2022) 36:377–87. doi: 10.1007/s40263-022-00904-6, 35380374 PMC8994715

[138] Roy A Stern T Harsh J Hudson JD Ajayi AO Corser BC . RESTORE: once-nightly oxybate dosing preference and nocturnal experience with twice-nightly oxybates. Sleep Med X. (2024) 8:100122. doi: 10.1016/j.sleepx.2024.100122, 39263597 PMC11388664

[139] Lavender M Martin C Anderson D. Sodium Oxybate: practical considerations and patient perspectives. CNS Drugs. (2025) 39:71–83. doi: 10.1007/s40263-024-01144-6, 40111740 PMC11950057

[140] Junnarkar G Allphin C Profant J Steininger TL Chen C Zomorodi K . Development of a lower-sodium oxybate formulation for the treatment of patients with narcolepsy and idiopathic hypersomnia. Expert Opin Drug Discov. (2022) 17:109–19. doi: 10.1080/17460441.2022.1999226, 34818123

[141] Wang YG Swick TJ Carter LP Thorpy MJ Benowitz NL. Safety overview of postmarketing and clinical experience of sodium oxybate (Xyrem): abuse, misuse, dependence, and diversion. J Clin Sleep Med. (2009) 5:365–71. doi: 10.5664/jcsm.27549, 19968016 PMC2725257

[142] Burgess CR Scammell TE. Narcolepsy: neural mechanisms of sleepiness and cataplexy. J Neurosci. (2012) 32:12305–11. doi: 10.1523/JNEUROSCI.2630-12.2012, 22956821 PMC3449314

[143] Kallweit U Bassetti CL. Pharmacological management of narcolepsy with and without cataplexy. Expert Opin Pharmacother. (2017) 18:809–17. doi: 10.1080/14656566.2017.1323877, 28443381

[144] Vignatelli L D'Alessandro R Candelise L. Antidepressant drugs for narcolepsy. Cochrane Database Syst Rev. (2008) 2008:CD003724. doi: 10.1002/14651858.CD003724.pub3, 18254030 PMC9030766

[145] Ristanovic RK Liang H Hornfeldt CS Lai C. Exacerbation of cataplexy following gradual withdrawal of antidepressants: manifestation of probable protracted rebound cataplexy. Sleep Med. (2009) 10:416–21. doi: 10.1016/j.sleep.2008.04.004, 18753005

